# Does awareness affect the restorative function and perception of street trees?

**DOI:** 10.3389/fpsyg.2014.00906

**Published:** 2014-08-15

**Authors:** Ying-Hsuan Lin, Chih-Chang Tsai, William C. Sullivan, Po-Ju Chang, Chun-Yen Chang

**Affiliations:** ^1^Laboratory of Healthy Landscape and Healthy People, Department of Horticulture and Landscape Architecture, National Taiwan University Taipei, Taiwan; ^2^Landscape Section, Urban and Rural Development Bureau, Taoyuan County Government Taoyuan, Taiwan; ^3^Department of Landscape Architecture, University of Illinois at Urbana-Champaign Champaign, IL, USA; ^4^Program of Landscape and Recreation, National Chung Hsing University Taichung, Taiwan

**Keywords:** DSBT, attention restoration, perceived restorativeness, manipulation on awareness degree

## Abstract

Urban streetscapes are outdoor areas in which the general public can appreciate green landscapes and engage in outdoor activities along the street. This study tested the extent to which the degree of awareness of urban street trees impacts attention restoration and perceived restorativeness. We manipulated the degree of awareness of street trees. Participants were placed into four groups and shown different images: (a) streetscapes with absolutely no trees; (b) streetscapes with flashes of trees in which participants had minimal awareness of the content; (c) streetscapes with trees; and (d) streetscapes with trees to which participants were told to pay attention. We compared the performance of 138 individuals on measures of attention and their evaluations of perceived restorativeness. Two main findings emerged. First, streetscapes with trees improved the performance of participants on attentional tests even without their awareness of the trees. Second, participants who had raised awareness of street trees performed best on the attentional test and rated the streetscapes as being more restorative. These findings enhance our knowledge about the role of an individual's awareness of restorative elements and have implications for designers and individuals who are at risk of attentional fatigue.

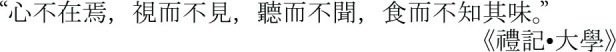

“*When one is absent-minded, he looks but sees nothing, hears but pays no attention, and eats but has no taste for it.*”

(The Great Learning, Classic of Rites)

## Introduction

Elements in the natural environment improve psychological well-being by reducing stress, restoring attention, and increasing positive emotions and esthetic values (Ulrich, 1984; Ulrich et al., 1991; Korpela et al., 2002; Groenewegen et al., 2006; Abraham et al., 2010; Bowler et al., 2010; Kaplan and Kaplan, 2011). Studies have demonstrated that increasing natural elements in an urban environment improved individuals' well-being (Kaplan, 2007; Chang et al., 2008; Korpela et al., 2010; Martens et al., 2011). In particular, natural elements in urban landscapes can help people pay attention or restore their capacity to pay attention. Kaplan and Kaplan's Attention Restoration Theory (ART) proposes that individuals' directed attention has limited capacity which becomes depleted when processing non-fascinating information about one's environment or performing attentionally demanding tasks (Kaplan and Kaplan, 1989).

Because directed attention is also needed for executive functioning and self-regulation (Korpela et al., 2001; Kaplan and Berman, 2010), directed attention fatigue can lead to a variety of negative consequences such as inability to concentrate, irritability, and even violent behavior (Kuo and Sullivan, 2001; Taylor and Kuo, 2009). Exposure to natural environments and to built environments that include natural elements such as trees have been shown to enhance an individuals' ability to recover from directed attention fatigue (Herzog et al., 2003; Laumann et al., 2003; Berto, 2005; Berman et al., 2008; Staats et al., 2010). Thus, understanding nature's ability to restore directed attention is useful in preventing the negative consequences of directed attention fatigue.

Do natural environments benefit individuals even when they are not aware of their surroundings? Kaplan (2001) argued that these restorative effects could happen without awareness. However, some studies used ART as a theoretical framework to discuss restorative environmental characteristics via self-rating questionnaires (e.g., Hartig et al., 1997, 2003; Chang et al., 2008), which requires participants to think back on their experience, hinting that restorative outcomes require awareness. Therefore, these studies implicitly assume positive environmental characteristics were consciously, not subconsciously, noticed by people. In one study, self-rated restorativeness was correlated with directed attention restoration (Berto, 2005). In spite of these associations, few previous studies have manipulated levels of awareness of natural elements and then examined the resulting impacts on attentional functioning. By manipulating people's awareness of natural elements, this study helps fill a gap in our knowledge regarding the extent to which awareness of natural elements in the landscape enhances one's restorative experience.

Our research is focused on urban streetscapes because the general public encounters urban streetscapes frequently. Few previous findings regarding the restorative effects of natural elements, such as trees, have specifically focused on urban streetscapes. Therefore, we raise two research questions: Will adding natural elements (e.g., trees) into a streetscape enhance restorative effects? Will the effects differ depending on the level of awareness that participants have of the natural elements? By answering these questions we hope to better understand the role of awareness in the restorative effect of nature. Our findings will have implications for educators, health care providers, designers, and urban planners who want to enhance the restorative effects of urban landscapes.

### Restorative effects of nature in urban environments

Kaplan's (1995, 2001) ART proposes that attention consists of two components: “*Involuntary attention*, where attention is captured by inherently intriguing or important stimuli, and *voluntary* or *directed attention*, where attention is directed by cognitive-control processes” (Berman et al., 2008). Directed attention fatigue can be relieved in a green setting because such places are often softly fascinating and thus allow a person to give their directed attention a rest (Herzog et al., 1997; Berto et al., 2008). An extensive body of empirical evidence has accumulated in support of ART. The findings come from very green settings such as large and small forests (Park et al., 2010; Shin et al., 2010), rural areas (Roe and Aspinall, 2011), wilderness settings (Hartig et al., 1991), and prairies (Miles et al., 1998). But the same is true for more modestly green settings such as community parks (Hartig et al., 2003; Krenichyn, 2006; Fuller et al., 2007; Korpela et al., 2008), schools (Matsuoka, 2010), and neighborhoods (Tennessen and Cimprich, 1995; Wells, 2000; Kuo and Sullivan, 2001; Taylor et al., 2002; Rappe and Kivelä, 2005).

ART identifies four characteristics of physical settings that contribute to restorative experiences. The first characteristic is *fascination*, the first proposed and necessary component of a restorative setting, describes objects or places that require little or no attentional effort—that is, little or no directed attention. A gentle form a fascination, what Kaplan and Kaplan (1989) call soft fascination (e.g., watching a waterfall, leaves moving in a breeze, fish in a pond), holds your involuntary attention in such a way as to leave some capacity to examine some of the thoughts that have been running around in your head. Softly fascinating settings foster restorative experiences (Kaplan et al., 1998; Herzog et al., 2011).

*Being away* (Kaplan and Kaplan, 1989) involves eliminating distractions from your surroundings, taking a break from your usual work or responsibilities, and ceasing pursuit of attentionally demanding tasks or activities. You might experience a feeling of being away if you stayed all day in an elevator. But doing so would by unlikely to produce a restorative experience. The Kaplans suggest that a sense of extent might also help.

*Extent*, the third proposed component of a restorative setting, describes a place that is “rich enough and coherent enough so that it constitutes a whole other world” (Kaplan, 1995, p. 173). “In a coherent environment, things follow each other in a relatively sensible, predictable, and orderly way” (Kaplan, 2001, p. 488). Extent is also aided by a setting that has sufficient scope. The key with respect to scope is that the setting be either physically large enough or conceptually large enough that one's mind can wonder within it. This process of allowing your thoughts to drift away from your daily activities into something that is rich and non-threatening seems an important part of a restorative experience. In this study, we examine the extent in terms of the level of coherence and scope available in various streetscapes.

*Compatibility* is the final proposed component of a restorative setting. Compatibility refers to the extent to which an environment supports your inclinations and purposes. It involves the fit between what you are trying to accomplish in the moment and the kind of activities supported, encouraged, or demanded by the setting (Herzog et al., 2003). Some settings will work against one's inclinations, others will meet some of them, and still others will be supportive in most every way. Settings that contain natural elements are often compatible with the kind of activities that lead to restoration.

Several studies have used these four environmental characteristics to measure a landscape's restorative qualities (Hartig et al., 1997; Laumann et al., 2001; Herzog et al., 2003; Berto, 2005). In this study, we also ask participants to evaluate the landscapes according to these characteristics of restorative landscapes.

### Does the degree of awareness influence these restoration?

The role of awareness in understanding the impacts of green spaces on restoration is unclear. In previous studies, the role of voluntary attention and involuntary attention, directed attention and fascination, and selective attention have been examined. Little research, however, has examined the extent to which paying attention to landscape features impacts attentional restoration. Some finding suggests that an environment can affect perceptions without awareness. For example, when it comes to people-environment relationships, the interaction is assumed to be a preconscious process (Parsons and Daniel, 2002). However, Kaplan (2001) suggested that paying attention to landscape characteristics might lead to enhanced restoration. The effect of participants' awareness of landscape features on restorative outcomes has yet to be tested and clarified.

During the process of visual perception, visual attention and awareness are inseparable. When individuals receive visual information, their visual attention focuses on specific bits of information and inhibits their awareness of other information. Visual awareness extracts the targeted information and sends it to the brain for further interpretation (Goldstein, 2007, p. 5).

Can different levels of visual awareness be observed and manipulated in experiments? Yes, research demonstrates that awareness levels can be manipulated (Tang and Posner, 2009). Subliminal visual attention, in which participants see something for such a short time that they are not fully aware of it, can be simulated in a laboratory setting (Erdelyi, 2004). People who were shown rapid images of snakes and spiders had the same level of fear as individuals who were shown the same images for longer periods of time. This suggests that attentional response can be activated very rapidly and without heightened awareness, requiring only minimal stimulus input (Globisch et al., 1999).

Visual awareness can be heightened when participants are instructed to carefully investigate a setting (Leff et al., 1974; Duvall, 2011). Works by Duvall (2011, 2012, 2013), for example, showed that instructions provided in a walking activity could alter perceptions and satisfaction of the environment and hence change the walking behavior. This suggests that awareness of the vegetation in a setting, in contrast to visual attention, could be heightened through instructions. If the awareness of the vegetation is strategically heightened, individuals may experience the landscape differently and report enhanced psychological well-being.

Although ART proposes that viewing natural elements such as trees, water, and flowers can restore directed attention, exposure to specific features in a setting and the awareness of those features have not been examined. We are therefore curious about the extent to which awareness levels influence attention restoration and the evaluation of the restorative characteristics of a setting—being away, fascination, extent (including scope and coherence), and compatibility. In sum, we ask two questions: To what extent is the greenery of an urban street beneficial to the attention-restoration experience? Will the degree of awareness of the streetscape's green features impact directed attention restoration and people's evaluation of an environment's restorativeness?

## Method

The objective of this study is to investigate whether directed attention and the evaluation of a streetscape's restorative characteristics would be affected by different degrees of awareness. To achieve this goal, we exposed 138 participants to four different conditions: 1. Urban streetscapes with no greenery, 2. Urban streetscapes with brief flashes of greenery (enough to direct attention but not awareness), 3. Urban streetscapes with greenery presented the entire time, and 4. Urban streetscapes with the same greenery as in the third manipulation but with added instructions to pay attention to the greenery. Participants were randomly assigned to view one of the four conditions and were asked to rate the four restorative characteristics and complete tests to measure how their directed attention capability recovered.

### Participants

A total of 140 undergraduate students from National Taiwan University (NTU) participated in this study. After eliminating two survey questionnaires with outlier total scores, the final sample is 138, consisting of 73 females and 65 males. The experiments were carried out between May and June in 2012 in classroom contexts in the Department of Horticulture and Landscape Architecture at NTU. The purpose of the study was explained at the beginning and the students were free to leave or to participate. The students who volunteered to participate received no compensation or credit for their participation.

### Stimulus materials

**Figure 1:**
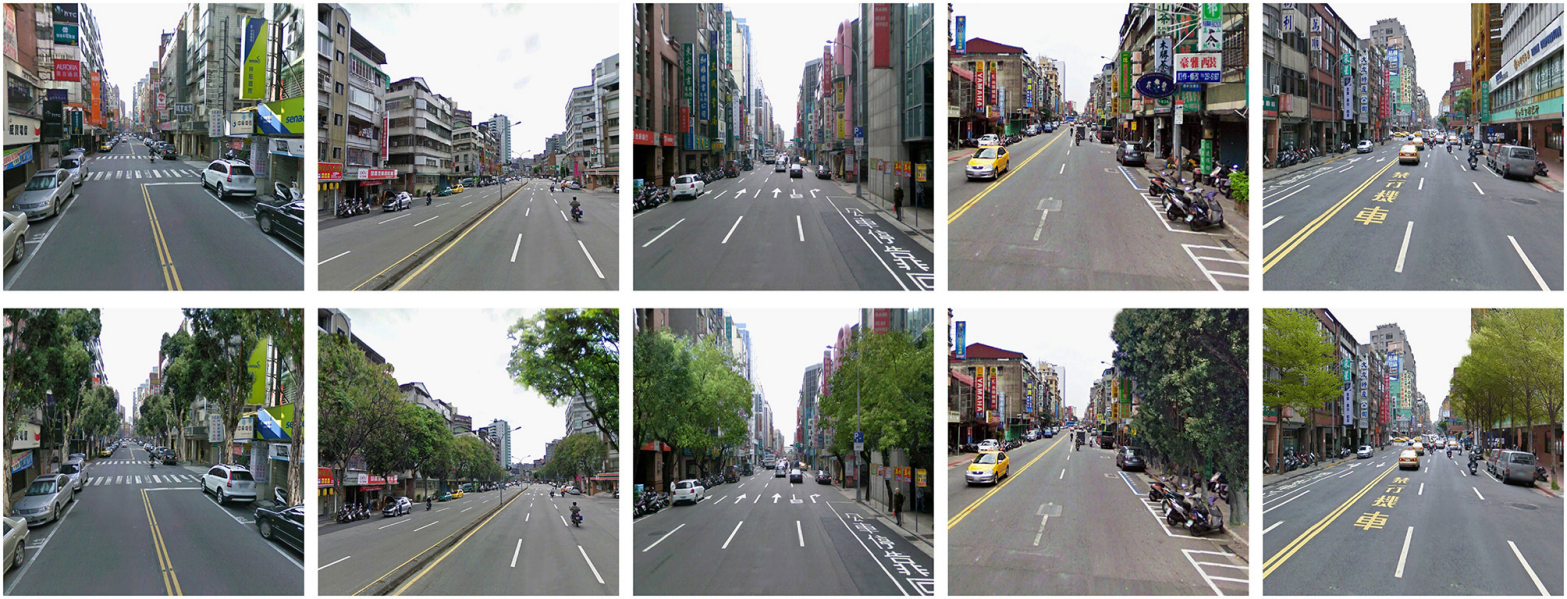
**Streetscape images**. (Upper row, streetscape with no greenery; Lower row, streetscape with simulated greenery).

To create the streetscapes, we took photographs of mixed residential-commercial areas with buildings between four and six-stories tall. Photographic images of five streetscapes without greenery in Taipei City were taken, which were used as the stimulants in the No Trees group. These images were taken at a height of 1.5 meters. Next, we used Adobe Photoshop CS 3.0 to edit the images to simulate streetscapes with 30–40% greenery along the sidewalks (Figure 1). The streetscapes with trees were used as the stimulants for the three groups with the same amount of greenery but different levels of awareness.

### Treatment conditions

Following the between-subject design, participants were randomly assigned to one of four groups with differing greenery images and awareness levels: (a) The No Trees group was shown streetscape images with no greenery. The awareness level for this group was none. (b) The Minimal Awareness group was shown the same images but mixed with rapid flashes of simulated greenery a few times. Participants could notice the trees flashing in and out, but were not substantially aware of the greenery. (c) The Moderate Awareness group was shown the images with simulated greenery for the entire time rather than in brief flashes. The awareness level for this group is moderate. (d) The Heightened Awareness group was shown the same greenery images as the previous two groups but was also given verbal instruction to observe the greenery carefully. Therefore, their awareness of the greenery was heightened.

**Figure 2:**
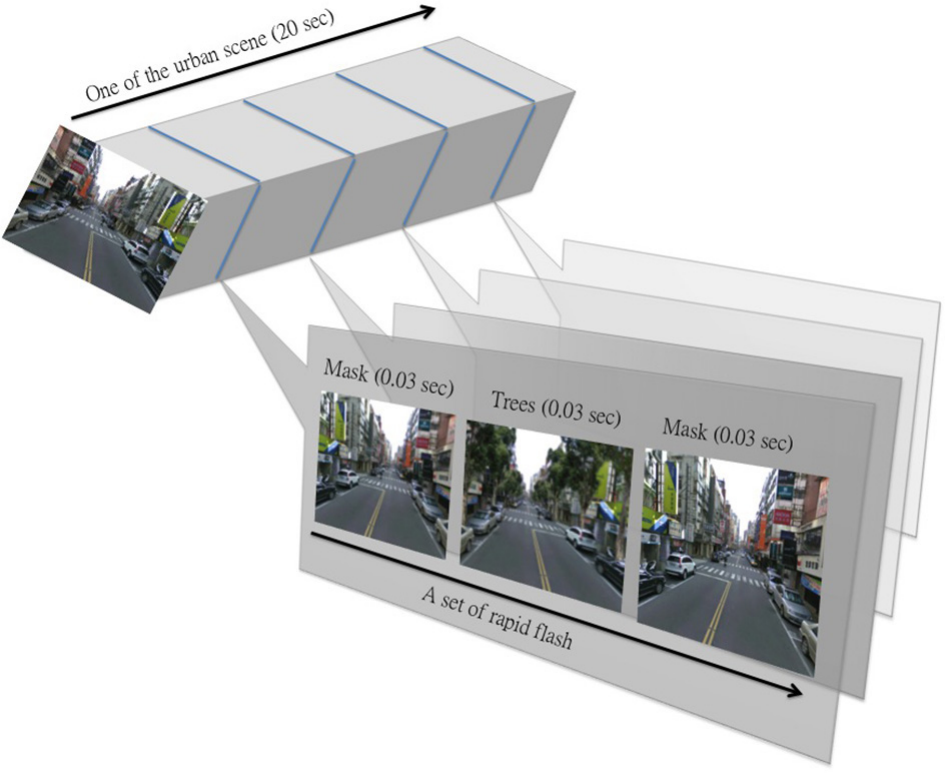
**How the rapid flashed of street trees were flashed in**. (Note: A mask is the horizontally flipped image of the streetscape being played).

Streetscapes for each group consisted of five images. Each image was shown for 20 s (100 s in total). Prior to seeing these streetscape images, there was a slide with these instructions: “*Now a number of images of streetscapes will be shown. Please relax and imagine being in the settings. You will be asked to answer some questions about the environments after watching these images. Please observe them closely.*” In the Heightened Awareness group, this last sentences was changed to “Please observe the plants closely.” The No Tree participants saw five images of the streetscape without greenery. For the Minimal Awareness group, while the participants were watching the original streetscape images, flashes of images containing greenery appeared every 5 s. Each flash was composed of three images: (a) a horizontally flipped image of the same streetscape image (mask), (b) a streetscape image with added greenery, and (c) a horizontally flipped image of the same streetscape image (mask) (see Figure 2). These three images appeared for 0.03 s each. The participants in this group saw these flashes of images without seeing actual content of trees. For the Moderate Awareness group, participants saw the images of the streetscapes with greenery present the entire time. The Heightened Awareness participants saw the images of the streetscapes with greenery and received these instructions: “*Now a number of images with plants will be shown. Please relax and imagine being in these settings. You will be asked to answer some questions about the plants after watching these images. Please observe the plants closely.”*

### Procedure

The experiments were scheduled with students in one of several classrooms directly following the class. Participants had just finished a class and were in a slightly fatigued state. We explained the purpose of the experiment, which was to understand how viewing a landscape would affect their responses and feelings. We then explained the experimental procedure to the participants. Next, we showed an example image (also from the street in Taipei, but not the image used in the stimulus material) to ensure that every participant could see the screen clearly. Then, participants engaged in a Digit Span Backward Test (DSBT), to evaluate their level of directed attention. The procedure for this test is described in Section Attention task.

The experiment started with the DSBT pre-test, after which the participants were shown five sets of images of streetscapes. Participants were then asked to answer five Perceived Restorativeness Scale (PRS) questions, and then underwent the DSBT post-test. The DSBT prompt and the images of the streetscapes were presented on a screen with PowerPoint 2007. At the end of these tests, participants answered demographic questions and were then asked to identify the species of the plants they had just seen (open-ended questions). The full procedure took 25–30 min to complete.

### Measurements

#### Attention task

This study employed the DSBT to measure directed attention before and after watching the stimulus so we were able to determine changes in attentional functioning. In the DSBT test, participants must exercise their short-term memory, holding in memory—then repeating in reverse order—a string of numbers. Prior to the test, we gave participants the following instructions: “You will see some numbers appear on the screen, each number will appear for 1 s and then another number will appear. Read the numbers carefully. After the numbers have been presented, write them down in reverse order. The task will last for 14 trials. You can stop whenever you can't remember the sequence.” We began with a practice trial after which participants were allowed to ask questions. After we addressed any questions, the real trials began. Each trial began with a cross in the middle of the screen and ended with a word “answer.” Only when the instructor confirmed the participant has looked back to the screen then did the next trial begin. The length of the set of numbers gradually increased from 4 to 10 digits; two sets of numbers were presented at each length, such that there were two sets of four digits, two at five digits and so fourth for a total of 14 trials. The length of digits repeated correctly just prior to the first mistake the participant made was used as the test score (a minimum score of 4 and a maximum score of 10).

#### Perceived restorativeness

Based on ART's characteristics of a restorative environments, participants rated five environmental characteristics (i.e., being away, fascination, coherence, scope, and compatibility) on a seven-point scale (0 = strongly disagree, 3 = neither agree or disagree, 6 = strongly agree) which was translated from Berto's 5-item version of PRS (Berto, 2005). Translated Mandarin version of the 5-item statements is presented in the Appendix. These five items were:

- That is a place which is away from everyday demands and where I would be able to relax and think about what interests me (being away);
- That place is fascinating; it is large enough for me to discover and be curious about things (fascination);
- That is a place where the activities and the items are ordered and organized (coherence);
- That is a place that is very large, with no restrictions to movements; it is a world of its own (scope);
- In that place, it is easy to orient and move around so that I could do what I like (compatibility).

#### Reported plant species

The final item in the questionnaire asked participants to identify the different plant species they had seen. For a manipulation check, this question is expected to test how much participants were aware of the greenery, depending on their awareness group. We hypothesized that the greater the awareness level, the greater the number of reported plant species.

## Results

### Manipulation check—reported plant species

**Figure 3:**
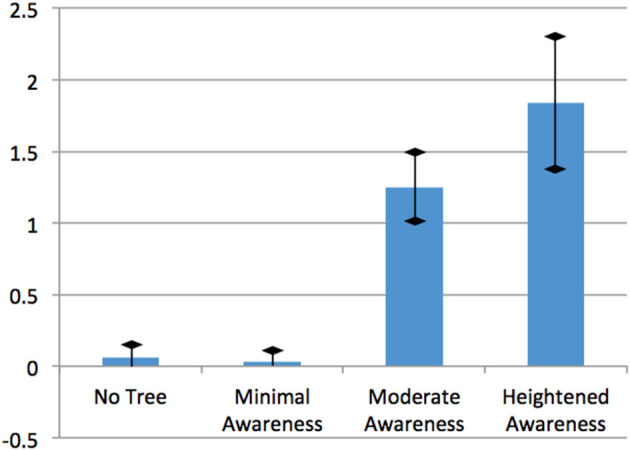
**The reported number of plant species by each group**. The error bars show a 95% confidence interval.

Were we able to successfully manipulate the level of awareness for the different groups? In order to verify the effects of the awareness level manipulation, we examined the difference between the reported numbers of plant species. Results showed significant differences between the reported number of plant species by the four groups [*F*_(3)_ = 47.64, *p* ≦ 0.001]. Through Scheffe's *post-hoc* analysis we confirmed that there is no difference between the reported number of plant species between the No Tree group, the group exposed to urban cityscapes with no trees (mean = 0.06; *SD* = 0.24), and the Minimal Awareness group, the group shown brief flashes of trees (mean = 0.03; *SD* = 0.18). The reported number of plant species by the Moderate Awareness group, the group shown constant images of cityscapes with trees (mean = 1.25; *SD* = 0.73) is greater than that by No Tree and Minimal Awareness groups. The number of species reported by the Heightened Awareness group, the group shown images of streetscapes with trees and given verbal instruction to pay close attention to the greenery (mean = 1.84; *SD* = 1.26), is greater than all other groups (Figure 3). These outcomes confirmed that the manipulation of awareness level for the groups produced the expected effects.

### Greenery and directed attention recovery

To ensure that there is no difference between the cognitive competencies of the test subjects in the four different groups, we used ANOVA to test the pre-test scores of DSBT, the attentional test. The result showed that there is no difference of the subjects from the four groups [*F*_(3)_ = 1.97, n.s.].

In order to confirm that looking at landscape photos (the treatment in the experiment) affects participants' ability to focus their attention, we conducted a paired *t*-test on the pre-test and post-test scores of the DSBT. Results demonstrated that there are significant differences between the pre-test and post-test scores of all the four groups (Table 1), which indicates that the treatments employed generated the expected outcomes. The post-test scores achieved by the No Tree group are noticeably lower than the scores on their pre-test. In other words, the directed attention of these participants did not recover during the process of looking at the photos; rather, it diminished. On the other hand, the post-test scores achieved by the other three groups (i.e., Minimal Awareness, Moderate Awareness, and Heightened Awareness) are all significantly greater than their respective pre-test scores. That is, the appearance of greenery, regardless of whether the participant was aware of the shape and type of the plant, has restorative effects on directed attention fatigue (Table 1).

**Table 1:** **Mean directed attention score before and after treatment**.

**Group**	**DSBT pre-test**	**DSPT post-test**	***T***
No tree (*n* = 34)	7.12 (1.45)	6.53 (1.38)	2.385^*^
Minimal Awareness (*n* = 31)	6.45 (1.55)	7.06 (1.44)	−2.31^*^
Moderate Awareness (*n* = 36)	6.28 (1.50)	6.83 (1.44)	−2.28^*^
Heightened Awareness (*n* = 37)	6.57 (1.56)	8.05 (1.53)	−5.68^**^

*Standard deviations are in the parentheses.*

*Note:*

* *p ≦ 0.05,*

** *p ≦ 0.001.*

### Awareness and directed attention recovery

In order to identify whether the degree of awareness of streetscape greenery affects the recovery of directed attention, the next step is to test for differences across these four groups. We first calculated the difference between pre-test and post-test scores of the DSBT, the directed attention test (Table 2); next, we employed ANCOVA to examine the differences between the test scores of the four groups. The results showed that awareness level does have an effect beyond and above the attention recovery level (difference between DSBT pre-test and post-test) even after we controlled the participant's baseline (pre-test), where *F*_(3, 133)_ = 11.84, *p* < 0.001. Paired comparison showed that the attention recovery score achieved by the Heightened Awareness treatment is significantly greater than that achieved by all the other groups. There is no difference between the Moderate Awareness and the Minimal Awareness treatments, and both scores are higher than that achieved by the No Tree treatment.

**Table 2:** **Cognitive performance of the four test groups**.

	**No tree**	**Minimal awareness**	**Moderate awareness**	**Heightened awareness**	***F***
DSBT^a^	−0.59^+^^◈^(1.44)	0.61^+^^||^ (1.48)	0.56^+^^||^ (1.46)	1.49^◈^^||^ (1.59)	11.84^***^
PRS^b^	13.59^+^ (4.05)	14.90 (3.70)	15.31 (4.41)	16.81^||^ (5.86)	2.92^*^
Being away	2.32 (1.07)	2.29 (0.94)	2.83 (1.38)	3.03 (1.34)	3.21^*^
Fascination	2.82 (1.29)	3.10 (1.08)	2.97 (1.21)	3.14 (1.32)	0.45
Coherence	2.97^+^ (1.24)	3.52 (1.15)	3.61 (1.15)	4.05^||^ (1.33)	4.66^**^
Scope	2.35^+^ (1.04)	2.84 (1.29)	2.78 (1.07)	3.14^||^ (1.55)	2.31^+^
Compatibility	3.12 (1.27)	3.16 (1.21)	3.11 (1.43)	3.46 (1.56)	0.52

*Standard deviation in parentheses.*

+ *p ≦ 0.1,*

* *p ≦ 0.05,*

** *p ≦ 0.01,*

*** *p ≦ 0.001; a: The changes between pre-test and post-test were treated as dependent variables while the pre-test scores were controlled as covariants; b: Perceived Restorativeness Scale;*

+ *Significantly different from Heightened Awareness treatment;*

◈ *Significantly different from Minimal and Moderate Awareness treatment;*

|| *Significantly different from No Tree treatment.*

### Awareness and perceived restorativeness

To what extend did awareness of greenery influence participants' assessments of how restorative the street scenes were? To examine this question, we tested the groups' scores for the PRS. Results showed a significant difference between the total PRS scores (Table 2). The Heightened Awareness group rated the scenes as significantly more restorative than the No Tree group. However there was no other difference between groups. This outcome implies that the awareness enhancing strategies may play a role in perceiving or appreciating restorativeness of urban greenery (see Table 2).

Next, we focused on the characteristics evaluated in the PRS questions separately in order to see how levels of awareness influenced evaluation of each characteristic. As can be seen in Table 2, there are significant differences among the groups for “being away” and “coherence.” There is also a marginal difference among the groups for “scope.” Scheffe *post-hoc* analyses showed that the Heightened Awareness group rated the landscape characteristics “coherence” higher and “scope” marginally higher than No Trees group. The “being away” characteristic is not different among the groups. There is no other difference among the groups. These findings suggest that heightened awareness made participants more likely to notice and appreciate the sense of coherence and scope in the streetscapes.

## Discussion

This study examined the extent to which raising awareness about trees in a streetscape could impact a person's capacity to pay attention and their evaluation of the restorativeness of a setting. The results revealed that increasing awareness had systematically significant impacts on participant's scores assessing their capacity to direct their attention. The findings provide new information about the benefits of green infrastructure on an individual's capacity to pay attention, shed new light on the role of awareness of one's surroundings, and raise questions for future research.

### Benefits of urban street trees on attention

The results confirmed previous research demonstrating that views of trees have positive impacts on adult's capacity to pay attention. We measured the attention performance with the DSBT before and after treatment for all four groups. As expected, all three groups that had views of street trees, regardless of the level of awareness that participants had of the trees, improved their directed attention. Only the group that viewed street scenes without trees had a measurable decrease in attentional functioning.

This contrasting result of decreased attention performance shown by the group looking at images of street scenes without trees demonstrated that built environments without vegetation did not possess restorative characteristics and might in fact drain existing reserves of attention. This finding is similar to the concept proposed by Parsons et al. (1998) that built elements do not produce positive effects on stress recovery and may lead to negative effects. Our findings are consistent with a study in which participants walked in an urban commercial district and in an arboretum. The authors of that study found that urban settings require people to constantly use directed attention to overcome the stimulants in their environments (Berman et al., 2008).

### Effect of awareness on attention and perceived restorativeness

The findings of this study confirmed the hypothesis that raising awareness of trees improved directed attention recovery and perceived restorativeness.

In terms of directed attention, our results demonstrated that even individuals who had minimal awareness of the trees (those who saw the trees as quick flashes) improved their scores on the test of direct attention. In other words, the appearance of natural content can produce restorative effects for attention restoration without awareness. Still, the participants who were instructed to purposely observe the trees performed even better on the directed attention tasks than those who were not instructed to pay attention to them. Our results resonate with research suggesting that nature content impacts people without their own awareness, but the effect will be greater if it is combined with a mental or emotional activity that increases awareness (Kaplan, 2001; Korpela et al., 2002).

Regarding to the evaluation of the PRS, individuals who were told to observe the trees rated the landscape scenes higher for their sense of coherence and marginally higher in terms of scope. These findings might suggest a viewer who is more aware of natural features such as trees may see the landscape more favorably from a restorative perspective (Kaplan, 2001).

Although the assessment of some restorative characteristics was increased slightly by heightened awareness, the effects did not emerge for “*fascination*” or “*compatibility.*” One possible reason that the awareness of street scenes with or without trees made no difference for the sense of *fascination* is that the images used in the experiment were quite familiar and common place to all participants. Perhaps trees that were more novel would have had an impact on fascination. Assessing such a possibility is an area for future research. The familiarity of the streetscapes may have also moderated the sense of compatibility. It is also possible that our measure of compatibility did not adequately capture the true meaning of the metric. In future research, it would be best to evaluate *compatibility*, with a more specific prompt than the one used here—one that focuses participants on their goals at the moment.

These findings demonstrate that when the participants' awareness of the street trees is heightened, they were better able to pay attention after exposure to a green setting than their counterparts who did not have their awareness heightened. These findings resonate with Duvall's (2011), who had adults walk outdoors for 30-min three times per week. Participants were randomly assigned to either the standard walking group or a group that practiced awareness of their surroundings. After the 2-week experiment, individuals who practiced awareness of their surroundings scored significantly higher on multiple measures of psychological well being including their capacity to pay attention. In his awareness treatment, Duvall's participants focused on their senses, imaged a new job or role as they walked, made guesses or inferences about the setting, or could cast spells that changed the environment in some way or another. In our study, we asked participants to engage in an even less cognitively demanding task—we simply told them to pay attention to the vegetation along the street. That such a simple intervention could have such significant results suggests the power of directing one's awareness to aspects of the physical environment that are restorative.

These findings have broad implications. Urban planners, architects, and landscape architects can use these results to help create places that are more restorative for individuals. Beyond simply providing restorative settings, they can find creative ways to engage people to be aware of the restorative aspects of the built environment.

Educators, business professionals, hospital workers, and others whose work places great demands on their capacity to pay attention can also use these results. Through the simple act of being aware of trees, flowers, a water feature or other restorative elements in the built environment, individuals are likely to benefit to a greater extent from these elements than if they were to simply accept them as part of their surroundings.

### Limitations and future research

By using simulated trees in the images shown to the participants, we were able to compare the differences between streetscapes with and without trees. The photographs used in the current study were taken from real streetscapes in Taipei, which are representative of densely populated urban environments. Future research should manipulate the density of the tree cover to see so that we might understand the interaction between a range of tree densities and heightened awareness.

Two other issues concern the expertise of our participants and their familiarity with the settings that were examined. Many of our participants had expertise in urban design, landscape architecture, or horticulture. Thus, they might not be representative of other adults who are not as focused on their physical surroundings. The interest of our participants in viewing green elements may be higher than that of untrained people, so the effect of green elements on their ability to recover their directed attention capacity might be enhanced compared to individuals who do not share these forms of expertise (Kaplan, 2001). Our participants were all students living in the city in which the pictures were taken. Some of the participants may have been familiar with the settings in the images. Future research should include participants from a variety of demographic backgrounds and education levels and also sample scenes with which none of the participants are familiar.

In this study, we found that awareness impacted attention. It seems possible that awareness of specific restorative elements in the landscape could also impact stress responses. A variety of studies have shown that exposure to natural elements in the landscape can help people recover from stressful events (Chang et al., 2008; Thompson et al., 2012; Tyrväinen et al., 2014). In these studies, awareness of the landscape was not a factor that was either manipulated or measured. To what extent does calling awareness to landscape features impact the speed of recovery from a stressful event? Given the results here, this is a question worthy of study.

Finally, more research should be done in real settings. In this study, we manipulated levels of awareness in a laboratory setting and found that awareness impacted attentional functioning. Future studies should manipulate levels of awareness in real-world settings as Duvall did (2011).

## Conclusion

Our study answers an important question: To what extent does the awareness of street trees impact the restorative impact of a street scene? Results are reassuring: The restoration of directed attention requires minimal awareness—so small, in fact that participants did not realize that the trees were present. Heightened awareness, however, further increased restoration of directed attention and influenced ratings of restorative quality of the street scenes. Awareness may not be a necessary part of the restoration process, but the evidence presented here suggests it can play a role in enhancing restorative experiences.

The practical benefits of this research are clear. Since contact with nearby nature benefits physical, psychological, and social well-being (Matsuoka and Kaplan, 2008; Barton and Pretty, 2010; Degenhardt and Buchecker, 2012; Thompson et al., 2012), the potential benefit of awareness clearly merits more attention. For individuals exposed to only minimal greenery in urban areas, heightened awareness may maximize the benefits of this minimal exposure.

Finding ways to encourage people to increase their awareness of restorative elements such as urban trees seems a worthy investment. Such an investment is likely to pay significant dividends for those of us in the world today who depend on our capacity to pay attention to meet our goals. That is to say, it is likely to pay dividends for most humans alive today.

### Conflict of interest statement

The authors declare that the research was conducted in the absence of any commercial or financial relationships that could be construed as a potential conflict of interest.
